# Prehistoric mitochondrial DNA of domesticate animals supports a 13th century exodus from the northern US southwest

**DOI:** 10.1371/journal.pone.0178882

**Published:** 2017-07-26

**Authors:** Brian M. Kemp, Kathleen Judd, Cara Monroe, Jelmer W. Eerkens, Lindsay Hilldorfer, Connor Cordray, Rebecca Schad, Erin Reams, Scott G. Ortman, Timothy A. Kohler

**Affiliations:** 1 Department of Anthropology, University of Oklahoma, Norman, Oklahoma, United States of America; 2 Laboratory of Molecular Anthropology and Ancient DNA, Washington State University, Pullman, Washington, United States of America; 3 Department of Anthropology, Washington State University, Pullman, Washington, United States of America; 4 Department of Anthropology, University of California, Davis, California, United States of America; 5 School of Biological Sciences, Washington State University, Pullman, Washington, United States of America; 6 Department of Anthropology, University of Colorado, Boulder, Colorado, United States of America; 7 Santa Fe Institute, Santa Fe, New Mexico, United States of America; 8 Crow Canyon Archaeological Center, Cortez, Colorado, United States of America; University of Florence, ITALY

## Abstract

The 13^th^ century Puebloan depopulation of the Four Corners region of the US Southwest is an iconic episode in world prehistory. Studies of its causes, as well as its consequences, have a bearing not only on archaeological method and theory, but also social responses to climate change, the sociology of social movements, and contemporary patterns of cultural diversity. Previous research has debated the demographic scale, destinations, and impacts of Four Corners migrants. Much of this uncertainty stems from the substantial differences in material culture between the Four Corners vs. hypothesized destination areas. Comparable biological evidence has been difficult to obtain due to the complete departure of farmers from the Four Corners in the 13^th^ century CE and restrictions on sampling human remains. As an alternative, patterns of genetic variation among domesticated species were used to address the role of migration in this collapse. We collected mitochondrial haplotypic data from dog (*Canis lupus familiaris*) and turkey (*Meleagris gallopavo*) remains from archaeological sites in the most densely-populated portion of the Four Corners region, and the most commonly proposed destination area for that population under migration scenarios. Results are consistent with a large-scale migration of humans, accompanied by their domestic turkeys, during the 13^th^ century CE. These results support scenarios that suggest contemporary Pueblo peoples of the Northern Rio Grande are biological and cultural descendants of Four Corners populations.

## Introduction

**Fig 1:**
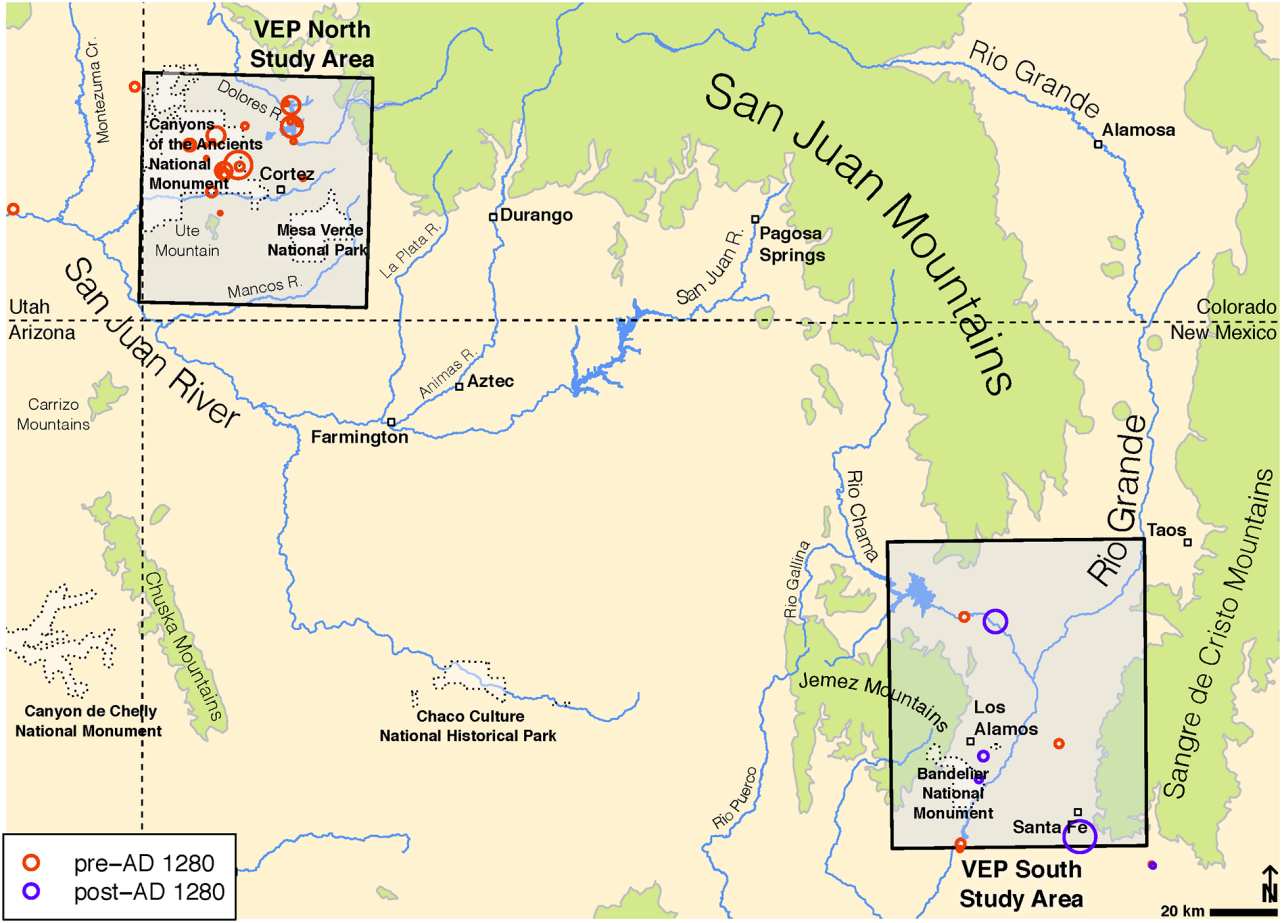
Location of the Central Mesa Verde (VEP North) and Northern Rio Grande (VEP South) study areas, and samples analyzed in this paper. Sizes of circles are proportional to sample sizes. Base map is derived from US Geological Survey Public Domain DEMs.

Although archaeologists have long recognized that maize farmers left the northern US Southwest by the late 1200s CE [1] the identities of the emigrating populations, their destinations, and the causes of the depopulation have been controversial. Recent work by the Village Ecodynamics Project (VEP) in the central Mesa Verde region (CMV) (Fig 1), the most populous portion of this vast area, has shown that population size peaked in the mid-1200s, was decreasing by no later than 1260 CE, and declined to zero by about 1280 CE [2].

Additional research [3, 4] has used evidence from biometry, linguistic prehistory, site architecture, etymology, and recorded social memory to argue that the ancestral Tewa population of the Northern Rio Grande (NRG) in New Mexico derives primarily (but not exclusively) from a migration of Pueblo III farmers from the CMV (Fig 1). However, discontinuities in many aspects of material culture between the pre-1280 CE CMV and post-1280 CE NRG [5] have led some to diminish both the size of the CMV influx and the importance of these immigrants for subsequent culture processes in the NRG [6, 7].

The tendency for populations of domesticated animals to migrate in tandem with human populations with whom they have a mutualistic relationship has been documented in previous studies (e.g., [8, 9–11]). Given current constraints on the acquisition of ancient DNA (aDNA) from human remains in the US Southwest, we consider patterns of aDNA variation from domesticated species as proxies for human population movements—and as particularly interesting populations to study in their own right [12–14].

Here, we investigate mtDNA haplotypic variation in domestic turkeys (*Meleagris gallopavo*) and dogs (*Canis lupus familiaris*) recovered from various archaeological sites to test whether populations of these animals were moved from the CMV (located in southwestern Colorado) to the NRG (located in north-central New Mexico) during the 13^th^ century CE (Fig 1). If ancestral Tewa populations (whose descendants still reside in the NRG) derive primarily from earlier CMV populations, one would expect mtDNA haplotype frequencies in turkey and dog remains from post-1280 CE NRG sites to be statistically distinguishable from earlier samples in the NRG. Further, if such differences are detectable, we would expect the new genetic variants in the post-1280 CE NRG to have been present in earlier CMV samples or to be derived from mitochondrial haplotypes observed in that region.

Patterns of usage for domestic turkeys in the two areas suggest the plausibility of this migration hypothesis. CMV populations greatly increased their use of domestic turkey in the 1000s CE as hunting pressure significantly depressed artiodactyl populations [2, 15, 16]. By Pueblo III times (1100–1280 CE) 64% of 90 faunal assemblages in the CMV have modified turkey index values >0.5 [15: 1835]. In the NRG, in contrast, domestic turkey had low dietary importance prior to 1280 CE; of the 23 assemblages tabulated by Akins [17: 218] only 6 (26%) have turkey indices exceeding 0.2. However, after 1280 CE half of the 18 tabulated assemblages have indices exceeding 0.5 in this same area [17: 218].

Given our observation that genetically-identified coyotes (*Canis latrans*) are present at six of the 14 archaeological sites from which we sampled canid remains (see below), we also evaluate whether dietary differences existed between coyotes and dogs, as ascertained by ratios of stable carbon (^13^C/^12^C) and nitrogen (^15^N/^14^N) isotopes preserved in the remains. Since we are investigating populations of domestic animals maintained by maize farmers, we expect dogs to exhibit enriched δ^13^C values over wild coyotes due to the domestic species having ready access to maize (a C4 plant) and/or domestic turkeys that depend largely on maize [18]. While this has not been previously addressed for prehistoric dogs in the US Southwest, both domestic turkeys (dating to approximately 620–1325 CE) and human farmers (dating from approximately 400 BCE to 1330 CE) have been demonstrated to exhibit similarly enriched δ^13^C values over wild captured turkeys, which had less access to carbon originating from C4 plants [18–20]. However, there is recent evidence for domestic turkeys on the fringe of the US Southwest that are not enriched in δ^13^C [21].

## Materials and methods

Turkey and canid remains were obtained from the collections of previously excavated archaeological sites. The remains were chosen as representatives of the domestic animals maintained by humans in the CMV and the NRG prior to the hypothesized migration (i.e., prior to 1280 CE), and those maintained in the NRG following the hypothesized migration (i.e., after 1280 CE). Specimen identification numbers and repositories for each sample are given in the supplemental information (S1 and S2 Tables).

### Samples from Southwest Colorado, pre-migration (predating 1280 CE)

Seventy-three turkeys were sampled from the following 11 sites: 1) Stanton’s Site (5MT10508, 1225–1260 CE, n = 4), 2) Woods Canyon Pueblo (5MT11842, 1140–1280 CE, n = 1), 3) 5MT1765 (600–1280 CE, n = 1), 4) Castle Rock Pueblo (5MT1825, 1260–1280 CE, n = 3), 5) Shields Pueblo (5MT3807, 1060–1260 CE, n = 28), 6) Shorlene's Site (5MT3918, 1180–1260 CE, n = 1), 7) Kenzie Dawn Hamlet (5MT5152, 1020–1260 CE, n = 1), 8) Yellow Jacket Pueblo (5MT5, 1100–1280 CE, n = 2), 9) Sand Canyon Pueblo (5MT765, 1225–1280 CE, n = 14), 10) Albert Porter Pueblo (5MT123, 1110–1250 CE, n = 12), and 11) Goodman Point Pueblo (5MT604, 1230–1270 CE, n = 6) (Table 1, S1 Table).

**Table 1:** Summary of turkey samples studied and the results of their genetic analyses. Sequences and mutational positions are relative to the turkey mtDNA reference sequence (EF153719; [30]). Only haplotypes of sequences spanning nps 15730–15973 are reported here. See S1 Table for full results of data collected in this study.

Unit of Comparison	Site	Site Name/Component	Date (CE)	n	Haplotypes of sequences spanning nps 15730–15973:				Partial Sequences	No DNA	Reference
					15735C, 15808C, 15953C (aHap1, aHap1?)	15782T, 15793T, 15808C, 15845T, 15953C (aHap2, aHap2?)	15782T, 15793T, 15845T, 15953C (aHap2b)	15793T, 15808C, 15953C (aHap2c)			
**CMV Pre-1280 CE**	5MT10508	Stanton's Site	1225–1260	4	4	0	0	0	0	0	This study
	5MT10508	Stanton's Site	1230–1270	5	5	0	0	0	0	0	Speller et al [22]
	5MT11842	Woods Canyon Pueblo	1140–1280	1	1	0	0	0	0	0	This study
	5MT1765	No name	600–1280	1	0	0	0	0	1	0	This study
	5MT1825	Castle Rock Pueblo	1260–1280	3	2	0	0	0	1	0	This study
	5MT1825	Castle Rock Pueblo	1250–1300	5	5	0	0	0	0	0	Speller et al [22]
	5MT3807	Shields Pueblo	1060–1260	28	22	0	0	0	4	2	This study
	5MT3807	Shields Pueblo	1020–1300	5	5	0	0	0	0	0	Speller et al [22]
	5MT3918	Shorlene's Site	1180–1260	1	0	0	0	0	0	1	This study
	5MT5152	Kenzie Dawn Hamlet	1020–1260	1	0	0	0	0	0	1	This study
	5MT5	Yellow Jacket Pueblo	1100–1280	2	1	0	0	0	0	1	This study
	5MT765	Sand Canyon Pueblo	1225–1280	14	8	1	0	0	3	2	This study
	5MT765	Sand Canyon Pueblo	1250–1280	5	5	0	0	0	0	0	Speller et al [22]
	5MT123	Albert Porter Pueblo	1110–1250	12	7	0	0	0	4	1	This study
	5MT123	Albert Porter Pueblo	900–1150	2	1	0	0	0	0	1	Speller et al [22]
	5MT123	Albert Porter Pueblo	1150–1300	6	6	0	0	0	0	0	Speller et al [22]
	5MT604	Goodman Point Pueblo	1230–1270	6	4	0	0	0	1	1	This study
	42SA22760	Hedley Ruin	1000–1300	6	5	1	0	0	0	0	Speller et al [22]
	42SA24756	Comb Wash	1150–1250	5	3	0	0	0	0	2	Speller et al [22]
	5MT23	Grass Mesa	600–920	1	0	1	0	0	0	0	Speller et al [22]
	5MT2854	Aldea Sierritas	720–800	2	1	1	0	0	0	0	Speller et al [22]
	5MT2151	LeMoc Shelter	720–900	1	1	0	0	0	0	0	Speller et al [22]
	5MT4650	Hanging Rock	720–880	2	2	0	0	0	0	0	Speller et al [22]
	5MT2181	Hamlet de la Olla	780–920	1	0	1	0	0	0	0	Speller et al [22]
	5MT2320	House Creek Village	800–920	1	1	0	0	0	0	0	Speller et al [22]
	5MT4475	McPhee Village	820–980	3	2	0	0	1	0	0	Speller et al [22]
	5MT4126	Ida Jean Site	1050–1150	2	2	0	0	0	0	0	Speller et al [22]
	5MT2149	Escalante Pueblo	1075–1250	2	1	0	0	0	1	0	Speller et al [22]
	5MT948	Mockingbird Mesa	900–1350	6	6	0	0	0	0	0	Speller et al [22]
	5MT13795	Mockingbird Mesa	700–1100	1	0	1	0	0	0	0	Speller et al [22]
	5MT1602	Mockingbird Mesa	1150–1300	11	9	1	0	0	0	1	Speller et al [22]
	24SAA22674	Great Bluff House	900–1150	3	3	0	0	0	0	0	Speller et al [22]
	24SAA22674	Great Bluff House	1150–1300	4	4	0	0	0	0	0	Speller et al [22]
	**TOTAL**			**152**	**116**	**7**	**0**	**1**	**15**	**13**	
**NRG Pre-1280 CE**	LA265	No name	800–950	1	0	0	0	0	1	0	This study
	LA3333	No name	1170–1230	4	3	0	0	0	0	1	This study
	LA6169	No name	1200–1280	4	1	2	0	0	1	0	This study
	LA672	Forked Lightning	1175–1350	2	1	1	0	0	0	0	Speller et al [22]
	**TOTAL**			**11**	**5**	**3**	**0**	**0**	**1**	**2**	
**NRG Post-1280 CE**	LA275	Abiquiu Pueblo	1350–1450	2	2	0	0	0	0	0	This study
	LA12	Arroyo Hondo, component I	1310–1345	56	27	1	1	0	16	11	This study
	LA12	Arroyo Hondo, component II	1310–1345	11	3	0	0	0	4	4	This study
	LA12	Arroyo Hondo, component unknown	1310–1345	2	1	0	0	0	1	0	This study
	LA908	Tsama Pueblo	1425–1625	20	10	0	0	0	7	3	This study
	LA4618	No name	1275–1325	7	7	0	0	0	0	0	Speller et al [22]
	LA217	Rainbow House	1400–1600	4	3	0	0	0	0	1	Speller et al [22]
	LA625	South Pueblo, Pecos	1300–1840	2	1	0	1	0	0	0	Speller et al [22]
	**TOTAL**			**104**	**54**	**1**	**2**	**0**	**28**	**19**	

We also used previously collected mtDNA data from 79 turkeys from 20 sites relevant to our investigation [22]: 1) Stanton’s Site (5MT10508, 1230–1270 CE, n = 5), 2) Castle Rock Pueblo (5MT1825, 1250–1300 CE, n = 5), 3) Shields Pueblo (5MT3807, 1020–1300 CE, n = 5), 4) Sand Canyon Pueblo (5MT765, 1250–1280 CE, n = 5), 5) Albert Porter Pueblo (5MT123, 900–1150 CE and 1150–1300 CE, n = 8), 6) Hedley Ruin (42SA22760, 1000–1300 CE, n = 6), 7) Comb Wash (42SA24756, 1150–1250 CE, n = 5), 8) Grass Mesa (5MT23, 600–920 CE, n = 1), 9) Aldea Sierritas (5MT2854, 720–800 CE, n = 2), 10) LeMoc Shelter (5MT2151, 720–900 CE, n = 1), 11) Hanging Rock (5MT4650, 720–880 CE, n = 2), 12) Hamlet de la Olla (5MT2181, 780–920 CE, n = 1), 13) House Creek Village (5MT2320, 800–920 CE, n = 1), 14) McPhee Village (5MT4475, 820–980 CE, n = 3), 15) Ida Jean Site (5MT4126, 1050–1150 CE, n = 2), 16) Escalante Pueblo (5MT2149, 1075–1250 CE, n = 2), 17) Mockingbird Mesa (5MT948, 900–1350 CE, n = 6), 18) Mockingbird Mesa (5MT13795, 700–1100 CE, n = 1), 19) Mockingbird Mesa (5MT1602, 1150–1300 CE, n = 11), and 20) Great Bluff House (24SAA22674, 900–1150 CE and 1150–1300 CE, n = 7) (Table 1).

Ninety-eight canids were sampled from the following seven sites: 1) Albert Porter Pueblo (5MT123, 1060–1260 CE, n = 13), 2) Grass Mesa Village (5MT23, 725–980 CE, n = 24), 3) Shields Pueblo (5MT3807, 1060–1260 CE, n = 26), 4) Lillian’s Site (5MT3936, 1180–1225 CE, n = 1), 5) McPhee Pueblo (5MT4475, 725–980 CE, n = 31), 6) Yellow Jack Pueblo (5MT5, 1020–1280 CE, n = 1), and 7) Sand Canyon Pueblo (5MT765, 1225–1280 CE, n = 2) (Table 2, S2 Table).

**Table 2:** Summary of canid samples studied and the results of their genetic analyses. Sequences and mutational positions are relative to the dog mtDNA reference sequence (U96639; [34]). Only haplotypes of sequences spanning nps 15459–15691 are reported here. Number of samples identified as coyotes are indicated, regardless of the span of the sequences used for the identification. See S2 Table for full results.

Unit of Comparison	Site	Site Name/ Component	Date (CE)	n	Dog haplotypes of sequences spanning nps 15459–15691							Dog Partial Sequence	Coyote	No DNA
					15484G, 15627G, 15639A	15627G, 15639A	15484G, 15567A, 15627G, 15639A	15484G, 15627G, 15639A, 15727T	15484G, 15522C, 15523C, 15627G, 15639A	15625C, 15627G, 15639A	15484G, 15553G, 15627G, 15639A			
**CMV Pre-1280 CE**	5MT123	Albert Porter Pueblo	1060–1260	13	4	1	0	0	0	0	0	1	1	4
	5MT23	Grass Mesa Village	725–980	24	13	0	1	0	0	0	0	2	0	8
	5MT3807	Shields Pueblo	1060–1260	26	5	0	0	1	0	0	0	1	5	14
	5MT3936	Lillian's Site	1180–1225	1	1	0	0	0	0	0	0	0	0	0
	5MT4475	McPhee Pueblo	725–980	31	16	0	0	0	2	0	0	2	2	10
	5MT5	Yellow Jacket Pueblo	1020–1280	1	0	0	0	0	0	1	0	0	0	0
	5MT765	Sand Canyon Pueblo	1225–1280	2	0	0	0	0	0	0	0	0	0	2
	**TOTAL**			**98**	**39**	**1**	**1**	**1**	**2**	**1**	**0**	**6**	**8**	**38**
**NRG Pre-1280 CE**	LA265	No name	800–950	5	2	0	0	0	0	0	2	0	0	1
	LA3333	No name	1170–1230	5	5	0	0	0	0	0	0	0	0	0
	LA391	No name	1000–1100	1	0	0	0	0	0	0	0	0	0	1
	LA6169	No name	1200–1280	2	2	0	0	0	0	0	0	0	0	0
	**TOTAL**			**13**	**9**	**0**	**0**	**0**	**0**	**0**	**2**	**0**	**0**	**2**
**NRG Post-1280 CE**	LA275	Abiquiu Pueblo	1350–1450	4	0	0	0	0	0	0	0	0	4	0
	LA12	Arroyo Hondo, component I	1310–1345	10	0	0	0	0	0	0	0	0	5	5
	LA12	Arroyo Hondo, component II	1310–1345	2	0	0	0	0	0	0	0	0	1	1
	LA908	Tsama Pueblo	1425–1625	1	0	0	0	0	0	0	0	0	1	0
	**TOTAL**			**17**	**0**	**0**	**0**	**0**	**0**	**0**	**0**	**0**	**11**	**6**

### Samples from Northern Rio Grande, pre-migration (predating 1280 CE)

Nine turkeys were sampled from the following three sites: 1) LA265 (800–950 CE, n = 1), 2) LA3333 (1170–1230 CE, n = 4), and 3) LA6169 (1200–1280 CE, n = 4) (Table 1, S1 Table).

Previously collected, comparative mtDNA data from two turkeys recovered from Forked Lightning (LA672, 1175–1350 CE) were used in our analyses [22] (Table 1). Note that Speller et al. [22] dated this site to 1300–1840 CE, but we associate these samples with the pre-migration period based on the tree-ring data and pottery from this site [23:42].

Thirteen canids were sampled from the following four sites: 1) Peña Blanca (LA265, 800–950 CE, n = 1), 2) LA3333 (1170–1230 CE, n = 5), 3) Pojoaque (LA391, 1000–1100 CE, n = 5), and 4) Peña Blanca (LA6169, 1200–1280 CE, n = 2) (Table 2, S2 Table).

### Samples from Northern Rio Grande, post-migration (post-dating 1280 CE)

Ninety-one turkeys were sampled from the following three sites: 1) Abiquiu (LA275, 1050–1130 CE, n = 2), 2a) Arroyo Hondo (LA12 component 1, 1310-134CE 5, n = 56), 2b) Arroyo Hondo (LA12 component 2, 1370–1410 CE, n = 11), 2c) Arroyo Hondo (LA12 component unknown, 1310–1410 CE, n = 2), and 3) Tsama Pueblo (LA908, 1425–1625 CE, n = 20) (Table 1, S1 Table).

Previously collected, comparative mtDNA data from 13 turkeys recovered from three relevant sites were used in our analyses [22]: 1) LA4618 (1275–1325 CE, n = 7), 2) Rainbow House (LA217, 1400–1600 CE, n = 4), and 3) South Pueblo, Pecos (LA625, 1300–1840 CE, n = 2) (Table 1).

Seventeen canids were sampled from the following three sites: 1) Abiquiu (LA275, 1050–1130 CE, n = 4), 2a) Arroyo Hondo (LA12 component 1, 1310–1345 CE, n = 10), 2b) Arroyo Hondo (LA12 component 2, 1370–1410 CE, n = 2), and 3) Tsama Pueblo (LA908, 1425–1625 CE, n = 20) (Table 1, S1 Table).

### DNA extraction, polymerase chain reaction (PCR) amplification, and sequencing

All pre-polymerase chain reaction (PCR) activities were conducted in the Ancient DNA Laboratory at Washington State University (WSU), a laboratory located in a separate building from wherein PCR and post-PCR activities are conducted. The Ancient DNA Laboratory is a dedicated workspace for processing degraded and low copy number (LCN) DNA samples. Precautions aimed to minimize the introduction of contamination are practiced in the laboratory [24].

DNA was extracted from the turkey and canid remains by one or more of the following three methods. These methods were employed to aid in maximizing the success rate of recovering genetic information from these remains (e.g., [25]). Under the first extraction method (noted as Extraction Method 1 in S1 and S2 Tables), approximately 14–81 mg of material was carefully removed from the whole. These portions of bones or teeth were submerged in 6% (w/v) sodium hypochlorite (bleach) for 4 min [26] and the bleach poured off. The samples were then twice submerged in DNA-free water, with the water poured off following submersion. Samples were transferred to 1.5 mL tubes, to which aliquots of 500 μL of EDTA (ethylenediaminetetraacetic acid) were added, and gently rocked at room temperature for >48 hours. Samples were extracted in batches of 6–9 with 1–2 accompanying extraction negative controls (i.e., extractions to which no bone or tooth was added). DNA was extracted following the WSU method described by Cui et al. [27].

Under the second extraction method (noted as Extraction Method 1E in in S1 and S2 Tables), approximately 26–59 mg of material was carefully removed from the whole. These portions of bones or teeth were submerged in 6% (w/v) sodium hypochlorite (bleach) for 4 min [26] and the bleach poured off. The samples were then twice submerged in DNA-free water, with the water poured off following submersion. Samples were transferred to 1.5 mL tubes, to which aliquots of 500 μL of EGTA (ethylene glycol tetraacetic acid) were added, and gently rocked at room temperature for >48 hours. EGTA is an alternative decalcifying agent that has been demonstrated to be useful in the study of aDNA [28]. Samples were extracted in batches of seven with an accompanying extraction negative control. DNA was extracted following the WSU method described by Cui et al. [27].

Under the third extraction method (noted as Extraction Method 2 in S1 and S2 Tables), approximately 63–538 mg of material was carefully removed from the whole. The portions of bones or teeth were submerged in 6% (w/v) sodium hypochlorite (bleach) for 4 min [26] and the bleach poured off. The samples were then twice submerged in DNA-free water, with the water poured off following submersion. Samples were transferred to 15 mL tubes, to which aliquots of 2 mL of EDTA were added, and gently rocked at room temperature for >48 hours. Samples were extracted in batches of 6–9 with 1–2 accompanying extraction negative control. DNA was extracted following a modified protocol of Kemp et al. [29] described by Moss et al. [25].

DNA extracts were tested for the presence of co-extracted PCR inhibitors following Kemp et al. [28]. In the case that an extract was deemed to contain sufficient inhibitors to prevent possible amplification of either turkey or dog mtDNA (if present in an extract), it was subject to repeated silica extraction along with its accompanying extraction negative (to monitor inadvertent introduction of contamination). The DNA extracts were again tested for the presence of co-extracted PCR inhibitors. This procedure was carried out until the extracts were deemed to be inhibitor “free”. Refer to Kemp et al. [28] for a schematic illustration of the procedure.

Four overlapping amplicons (≤186 base pairs (bps) in length) were sequenced to cover a 460 base pair (bp) portion of turkey mtDNA from nucleotide positions (nps) 15554–16013, a part of the displacement loop, see [30], a comparable stretch of the genome investigated in previous studies [21, 22, 31] (Table 3). In the case that derived mutations were observed over those previously described for prehistoric Southwestern turkeys [22], re-amplification and re-sequencing was performed to confirm them, as they could either represent mutations that define novel lineages or be the result of postmortem nucleotide damage [32, 33]. Derived mutations that were not replicable were discounted as damage or error.

**Table 3:** Primers used to amplify turkey mtDNA, their coordinates relative to the turkey (EF153719; [30]) mtDNA reference sequence, amplicon lengths produced, and annealing temperatures. While primers were previously reported by Speller et al. [22], the coordinates of T15533F are corrected here, the target regions have been renamed, and the amplicon sizes are reported.

Target Region	Primer	Coordinates	Sequence (5' to 3')	Amplicon Length (bps)	Annealing Temperature
A1	T15533F	15533–15553	GTTGTTCTCAACTACGGGAAC	124	55°C
	T15656R	15634–15656	GTATGTGGTATATAAATGTATCG		
A2	T15612F	15612–15633	GGGGTATACTATGCATAATCGT	139	55°C
	T15750R	15730–15750	GTAGTCATAGGGAGAAATGG		
B	T15709F	15709–15729	ACGGACATAACAACCTTTACC	186	60°C
	T15894R	15875–15894	TCTGGTACGTCGAGCATAAC		
C	T15853F	15853–15874	CTTACTGTACTTACCCCATTTG	180	60°C
	T16032R	16014–16032	TCGACCGAGGAACCAGAGG		

To maximize comparability between turkeys maintained by humans in the CMV and the NRG prior to the hypothesized migration (i.e., predating 1280 CE), and those maintained in the NRG following the hypothesized migration (i.e., postdating 1280 CE), sequences were truncated to nps 15730–15973. Combined with previously collected (and similarly truncated) comparative data from Speller et al. [22], a total of 189 mtDNA sequences were used to compare turkeys from the three spatial/temporal units of analysis.

Six overlapping canid primer sets (≤202 bps in length), relative to the dog mtDNA reference sequence U96639 [34] and to the coyote reference sequence DQ480509 [35] were used to sequence canid mtDNA and spanned nps 15417–15775 (359 bps) for dogs and nps 15419–15773 (355 bps) for coyotes (Table 4). This stretch of the mitochondrial genome, beginning in the tRNA-Proline gene and ending in the control region [34], is comparable to that investigated in previous studies of prehistoric and extant American dogs (see studies summarized by [11]). One exceptionally common Southwest dog haplotype (derived by 15484G, 15627G, 15639A mutations) became apparent (Table 2) and replication efforts focused on verifying derived mutations from this general motif. All mutations derived from this haplotype were verified by ≥2 independent sequence reads. We used failure to confirm derived mutations as an indication that the mutation observed in a single read was most likely a product of postmortem nucleotide damage [32, 33]. Some of the same efforts permitted us to verify coyote haplotypes, but given that these animals were likely wild (based on their stable isotopic ratios of carbon and nitrogen), it was not our goal to authenticate the sequences from each of those samples, as they do not contribute to testing our main hypothesis about the movement of domesticate animals. To maximize comparability between sampled from the three spatial/temporal units of analysis, dog sequences were truncated to nps 15459–15691.

**Table 4:** Primers used to amplify dog and coyote mtDNA, their coordinates relative to the dog (U96639; [34]) and coyote (DQ480509; [35]) mtDNA reference sequences, amplicon lengths produced, and annealing temperatures.

Target Region	Primer	Coordinates to Dog mtDNA	Coordinates to Coyote mtDNA	Sequence (5' to 3')	Dog Amplicon Length	Coyote Amplicon Length	Annealing Temperature	Reference
DOG 1	D15401F	15401–15420	15403–15422	AAGCTCTTGCTCCACCATCA	195	191	60°C	This study
	D15595R	15571–15595	15569–15593	GATATAATATTATGTACATGCTTAT				
DOG A	D15421F	15421–15440	15423–15442	GCACCCAAAGCTGAGATTCT	197	193	58°C	Witt et al. [11]
	D15617R	15995–15617	15993–15615	GAGTTAATATGTCCTATGTAAGG				
DOG 2	D15534F	15534–15555	15532–15553	CTATGTACGTCGTGCATTAATG	178	178	60°C	This study
	D15711R	15692–15711	15690–15709	GGTTGATGGTTTCTCGAGGC				
DOG B	D15595F	15595–15617	15593–15615	CCTTACATAGGACATATTAACTC	202	202	60°C	Witt et al. [11]
	D15796R	15776–15796	15774–15794	AGAACCAGATGCCAGGTATAG				
COYOTE 1	C15401F	15399–15416	15401–15418	AGAAGCTCTTGCTCCACC	199	195	55°C	This study
	C15595R	15575–15597	15573–15595	AAGATATAATATTATGTACATGC				
COYOTE 2	C15551F	15553–15570	15551–15568	ATGGCTTGCCCCATGCAT	180	180	55°C	This study
	C15730R	15714–15732	15712–15730	AAGAGGGACATTACGAGCA				

PCR volumes were either 15 or 30μL. Fifteen microliter PCRs contained 0.32mM dNTPs, 1X Omni Klentaq Reaction Buffer, 0.24 μM of each primer, 0.3 U of Omni Klentaq LA, and 1.5 μL of template DNA. Thirty microliter PCRs contained the same except for 3 μL of template DNA. PCR cycling conditions consisted of: 1) a 3 min hold at 94°C, 2) 60 cycles of 15 s holds at 94°C, the annealing temperature (Tables 3 and 4), and 68°C, and 3) a 3 min hold at 68°C. Successful amplification was confirmed by separating 3–4 μL on 6% polyacrylamide or 2% agarose gels, which were stained with ethidium bromide and visualized under ultraviolet light. Amplicons were sequenced in both directions at either Elim Biopharm (Hayward, CA) or MC Lab (South San Francisco, CA). Sequencher (version 4.8) was used to align the sequences to comparative full mitochondrial genomes (turkey: EF153719 [30], dog: U96639 [34], coyote: DQ480509 [35]).

Fisher’s exact tests, conducted using the on-line calculator in VassarStats, were used to compare observed haplotype counts. An alpha level of 0.05 was set as the cut-off for statistical significance of the tests.

### Stable isotopic analysis

#### University of California-Davis

Subsamples of material from 23 canid remains were sent to the University of California-Davis (UCD) for stable carbon (^13^C/^12^C) and nitrogen (^15^N/^14^N) isotopic analysis. This was a blind test; whereas genetic species identification of these samples was known to the co-author who shipped the remains (B.M.K.), they were unknown to the co-author who processed them for stable isotopic ratios (J.W.E.).

Collagen was extracted by following a modified Longin procedure [36]. Bone samples were cleaned of any adhering soil using a small brush and dental pick, and the outer layer (approximately 0.5mm) removed using a Fordham microdrill. This step removed portions of the bone directly exposed to soil, and hence, most susceptible to diagenetic effects. The sample was then rinsed and sonicated in deionised water (dH_2_O) to remove any loose dust or other adhering material.

To isolate collagen, approximately 1g of cortical bone was demineralized with a solution of 0.5M hydrochloric acid (HCl) set in a refrigerator set to 5°C. HCl was replaced approximately at 48-hour intervals until the bone was soft and no longer visibly reacted with HCl solution, typically 1–2 weeks. The bone was rinsed three times with dH_2_O and soaked in 0.125M NaOH (sodium hydroxide) for 24 hours to remove humic acids and rinsed again in dH_2_O. To solubilize collagen, pH3 water was added to the vial and the sample placed in an oven set to 80°C. Every 24 hours, this water was removed, saved, and new pH3 water added until the bone completely solubilized or only residual bone powder remained, typically within 3 days. The vial containing the solubilized collagen was then centrifuged and the fluid pipetted into a clean vial and freeze-dried, isolating the collagen fraction.

δ^13^C_col_ and δ^15^N were measured by continuous-flow mass spectrometry (PDZ Europa ANCA-GSL elemental analyzer interfaced to a PDZ Europa 20–20 isotope ratio mass spectrometer) at the Stable Isotope Facility, University of California Davis. Carbon isotopes ratios, δ^13^C_col_, are reported in permil notation (parts per thousand) relative to the PeeDee Belemnite standard (arbitrarily set at 0‰), while N isotope ratios, δ^15^N, are expressed against N_2_ in modern atmospheric air (also arbitrarily set to 0‰). The long-term standard deviation for samples in the lab is 0.2‰ for δ^13^C and 0.3‰ for δ^15^N.

Sample quality was evaluated using collagen yield (% weight of mechanically cleaned bone) and atomic C/N ratios. Previous studies suggest that collagen yields greater than 2% produce radiocarbon dates within expected ranges [37]. Assuming similar diagenetic processes for non-radiocarbon (^13^C and ^12^C), we use this figure (≥ 2% collagen yield) as a cutoff for acceptable samples. Likewise, collagen samples with atomic C/N ratios lower than 2.9 or higher than 3.6 are typically considered degraded and unsuitable for archaeological interpretation [38, 39]. We use these same figures to indicate acceptable samples.

#### Washington State University

Subsamples of material from an additional 12 canid remains were processed for stable carbon (^13^C/^12^C) and nitrogen (^15^N/^14^N) isotopic analysis at WSU. This was also a blind test; genetic species identification of these samples was unknown to the co-author (L.H.) who processed them for stable isotopic ratios. Sample preparation used recently applied methodologies in current literature [40–42]. Approximately 200 mg of cortical bone were removed from the whole. The samples were washed with distilled (DI) water three times and submersed in 1 M HCl for 48 hours at 4°C for demineralization. After demineralization, the bone fragments were washed three times with DI water, and then submerged in 0.125 M NaOH for 24 hours at 4°C. The samples were again rinsed 3 times with DI water, and then were immersed in 2 mL of DI water and gelatinized for 24 hours at 90°C. The soluble “collagen” solution (supernatant) was removed, frozen, and lyophilized using Virtis FM5SL Freezemobile.

Approximately 1.25–1.5 mg of the collagen extracts were weighed (Mettler-Toledo #XP56DR), and run in an elemental analyzer (ECS 4010, Costech Analytical, Valencia CA) with a GC column and mass spectrometer to determine δ^13^C and δ^15^N ratios. Normalization of the samples was conducted with acetanilide and corn as quality controls. Standards for comparison of the generated values were C_VPDB_ (Vienna PeeDee Belemnite) for carbon and N_AIR_ (nitrogen content of the atmosphere) for nitrogen.

Once the isotopic readings were generated by isotope-ratio mass spectrometry (IRMS), raw delta values were regressed relative to the standard, to adapt the values to make them comparable to a standard (Eq 1). This was done using the raw delta equation:

$$\delta_{raw}=\left[\left(R_{sample}/R_{standard}\right)-1\right]*1000$$ (1)

where R _sample_ is the ratio of an element’s normal to heavy isotopes within the sample and R_standard_ is the ratio of an element’s normal to heavy isotopes within the standard. The regressed values were then used to create a line from which a linear equation was derived and used to calculate the standard deviations of the standards so the accuracy of the runs could be examined. The normalized delta values of the samples were then compared to the adapted standards using Eq 2:

$$\delta =\left[\left(R_{sample}/R_{standard}\right)-1\right]*1000$$ (2)

Two tailed t-tests were employed in StatPlus (AnalystSoft Inc.) to compare average δ^13^C and δ^15^N values, setting the alpha level at 0.05 for determining statistical significance of the tests.

## Results

One hundred and eighty DNA extractions were performed from 173 turkey samples (S1 Table). An average of 1.03 (SD 1.24) repeat silica extractions were required to sufficiently remove PCR inhibitors from these extractions. Complete or partial mtDNA sequences were recovered from one hundred and forty-four of the one hundred and seventy-three samples (83.2%) (S1 Table). It is notable that turkey mtDNA was neither observed in any extraction negative controls nor in any PCR negative controls.

Complete sequences ranging from nps 15554–16013 were obtained from twenty-three of the one hundred and forty-four of the samples (16%) that yielded analyzable mtDNA (S1 Table). Following established nomenclature [22], 19 of these turkeys belong to the aHap1 lineage, three to the aHap2 lineage, and one to the aHap2b lineage. Thus, 19 of 23 (82.6%) belong to the haplogroup H1 (of which aHap1 is a member), with the remaining four (17.4%) belonging to haplogroup H2 (of which aHap2 and aHap2b are members). Partial sequences from one hundred and four of the one hundred and forty-four samples (72.2%) could be used to confidently categorize individual turkeys as members of either the aHap1 or aHap2 lineage (noting that eventual resolution of these missing data may reveal them to belong to one or more derived lineages). These samples were recorded with question marks as “aHap1?” (n = 102) and “aHap2?” (n = 2) in S1 Table. Combining these complete and partial sequence data reveals that one hundred and twenty-one of the one hundred and twenty-seven samples (95.3%) belong to haplogroup H1 (i.e., aHap1 and aHap1? turkeys) and six of the one hundred and twenty-seven samples (4.7%) are members of haplogroup H2 (i.e., aHap2 and aHap2? turkeys) Partial sequences that do not permit confident assignment to either the aHap1? or aHap2? lineage or to haplogroup H1 or H2 were obtained from the remaining seventeen of the one hundred and forty-four samples (11.8%) that yielded analyzable mtDNA. Turkey mtDNA sequences have been deposited in Genbank (accession numbers MF197043—MF197186).

Our results are combined with those of Speller et al. [22] in Table 1, and Fisher’s exact tests of these combined results are given in Table 5. The tests reveal that mitochondrial haplogroup counts in the turkey populations from the CMV and the NRG prior to 1280 CE are significantly different (p = 0.0194) and are highly unlikely to represent a single panmictic population. In addition, there is a statistical difference in haplogroup frequencies between pre-1280 and post-1280 samples in the NRG (p = 0.0212); thus reflecting substantial change in haplogroup frequencies in NRG turkey populations over time. Finally, haplogroup frequencies among turkeys maintained in the pre-1280 CMV and the post-1280 NRG are practically identical (p = 1.0000). These results demonstrate that NRG turkey populations were initially distinct, but became indistinguishable from CMV turkey populations across the hypothesized period of migration from the CMV.

**Table 5:** P-values of Fisher’s exact tests used to compare haplogroup counts for turkeys across the three spatial/temporal units of analysis. Tests compare aHap1 and aHap2 frequencies from Table 1.

	Pre-1280 CMV	Pre-1280 NRG
Pre-1280 NRG	0.0194	
Post-1280 NRG	1.000	0.0212

One hundred and ninety-one extractions were performed across the 128 canid samples (S2 Table). An average of 0.43 (SD 0.84) repeat silica extractions were required to sufficiently remove PCR inhibitors from these extractions. Mitochondrial DNA was recovered from eighty-one of the one hundred and twenty-eight samples (63.3%). Of these 81 samples, 19 (23.5%) were identified as coyotes (Table 2, S2 Table). Coyotes were observed at three of the seven pre-1280 CE CMV sites (Albert Porter Pueblo, Shields Pueblo, and McPhee Pueblo). No coyotes were observed in the Northern Rio Grande samples pre-dating 1280 CE and all canid remains sampled from the post-1280 CE NRG (at Abiquiu Pueblo, Arroyo Hondo, and Tsama Pueblo) were identified as coyotes, despite the fact that several of these samples were originally recorded as “dog burials”.

**Fig 2:**
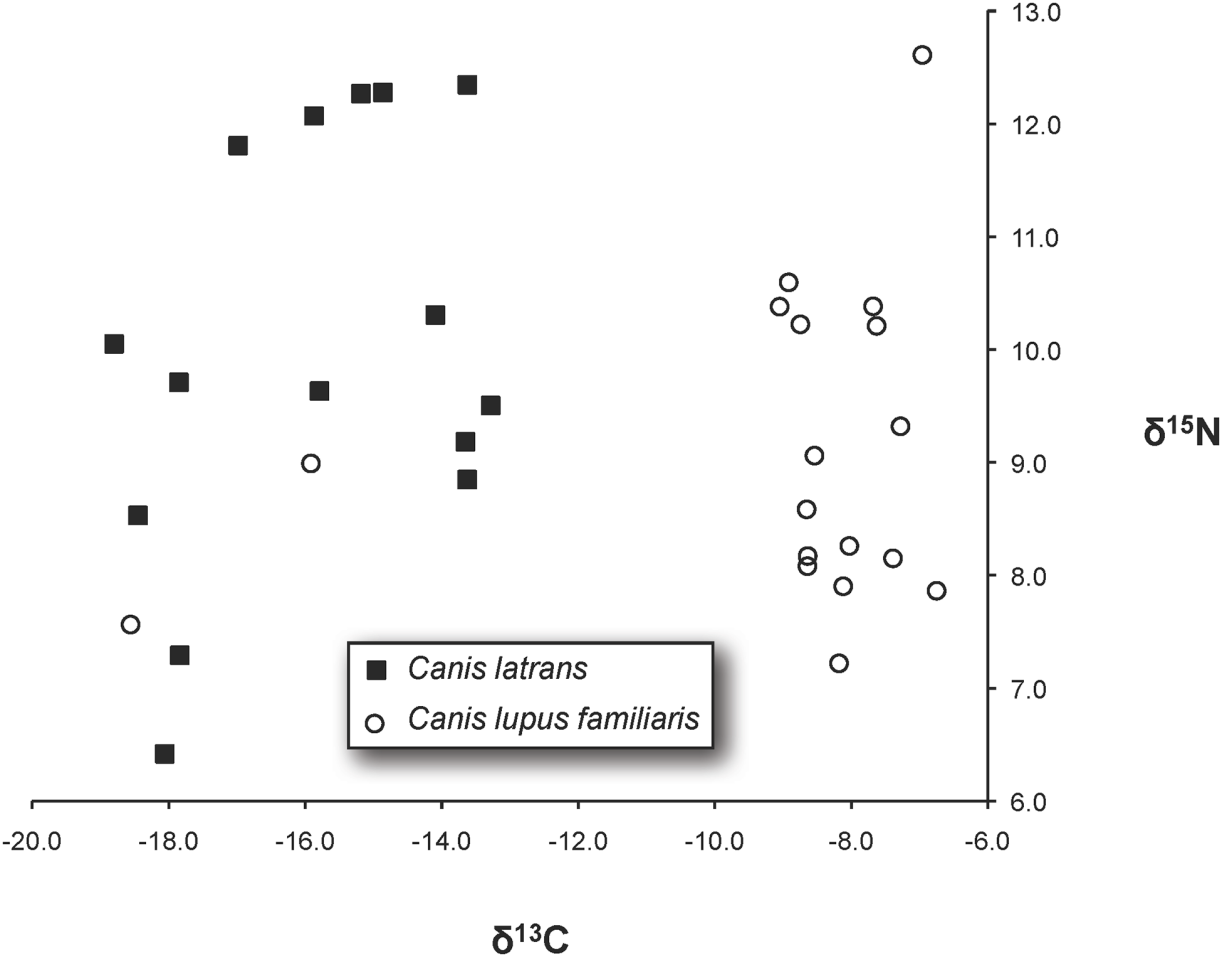
Plot of stable carbon (^13^C/^12^C) and nitrogen (^15^N/^14^N) isotope values observed from a subset of the canids for which genetic identification as dog or coyote was achieved. Dogs are indicated by white circles and coyotes as black squares. Values for each data point are reported in Table 7.

Stable isotopic data were generated for all 23 canid remains processed at UC Davis with C/N ratios of between 1.9 and 3.4 (Table 6). Two of the 12 samples, 5MT4475-10 and 5MT4475-16, processed at WSU yielded little collagen, produced unacceptable C/N ratios of 4.1 and 5.2, and were eliminated from the study. An average δ^13^C of -15.9 (SD 2.0) and δ^15^N of 10.0 (SD 1.9) was observed for the 15 coyotes samples and an average δ^13^C of -9.1 (SD 3.1) and δ^15^N of 9.1 (SD 1.4) was observed for the 18 dog samples. There is little overlap in the δ^13^C values for the two species (Fig 2), except for dog samples 5MT123-03 (δ^13^C: -18.6) and 5MT5-01 (δ^13^C: -15.9). Both of these dogs lived in the CMV prior to 1280 CE (S2 Table). With regards to 5MT123-03, only a rather short mtDNA sequence was generated (spanning nps 15441–15594), one that matches the reference sequence. However, lacking the 15484G mutations, this individual cannot belong to the most widespread dog haplotype observed in this study (i.e., 15484G, 15627G, and 15639A, see Table 2). While 5MT5-01 exhibits a unique haplotype (i.e., 15625C, 15627G, 15639A), it is one mutational difference (15625C) from a dog lineage observed at Albert Porter Pueblo (sample 5MT123-07). A two-tailed t-test indicates that δ^13^C average values of coyotes and dogs are indeed different (d.f. = 31, t-value = 7.31, p = 3.14x10^-8^). Based on dietary differences, these genetically identified coyotes were unlikely to have been domestic animals. Thus, only the hypothesized movement of dogs from the CMV to the NRG after AD 1280 was addressed.

**Table 6:** Results of the stable carbon (^13^C/^12^C) and nitrogen (^15^N/^14^N) isotopes ratios preserved in the subset of canid remains subjected to analysis. These data are also depicted in Fig 2.

Species	Laboratory Sample ID	Site	Site Name/Component	Date (CE)	Laboratory	Material Analyzed (mg)	d13C	d15N	C/N Ratio
Coyote	5MT123-08	5MT123	Albert Porter Pueblo	1060–1260	UCD	0.84	-17.9	9.7	3.3
	5MT3807-02	5MT3936	Lillian's Site	1060–1260	UCD	1.08	-13.6	12.3	3.3
	5MT3807-05	5MT3936	Lillian's Site	1060–1260	UCD	0.83	-15.2	12.3	3.3
	5MT3807-06	5MT3936	Lillian's Site	1060–1260	UCD	0.97	-14.9	12.3	3.3
	5MT3807-09	5MT3936	Lillian's Site	1060–1260	UCD	1.01	-18.8	10.0	3.3
	5MT3807-19	5MT3936	Lillian's Site	1060–1260	UCD	0.95	-17.0	11.8	3.3
	5MT4475-06	5MT4475	McPhee Pueblo	725–980	WSU	1.30	-15.87	12.07	3.3
	5MT4475-11	5MT4475	McPhee Pueblo	725–980	WSU	1.28	-18.45	8.53	3.2
	AB-01	LA275	Abiquiu	1350–1450	UCD	1.11	-13.3	9.5	3.3
	AB-02	LA275	Abiquiu	1350–1450	UCD	0.91	-13.7	9.2	3.3
	AB-03	LA275	Abiquiu	1350–1450	UCD	1.09	-13.6	8.8	3.3
	AH-03	LA12	Arroyo Hondo Component 1	1310–1345	UCD	0.89	-15.8	9.6	3.3
	AH-08	LA12	Arroyo Hondo Component 1	1310–1345	UCD	1.18	-18.1	6.4	3.3
	AH-12	LA12	Arroyo Hondo Component 2	1310–1345	UCD	1.11	-17.8	7.3	3.4
	TP-01	LA908	Tsama Pueblo	1425–1625	UCD	1.04	-14.1	10.3	3.3
						**Average**	-15.9	10.0	
						**SD**	2.0	1.9	
Dog	5MT123-01	5MT123	Albert Porter Pueblo	1060–1260	UCD	1.12	-7.7	10.4	3.3
	5MT123-03	5MT123	Albert Porter Pueblo	1060–1260	UCD	1.20	-18.6	7.6	3.3
	5MT123-04	5MT123	Albert Porter Pueblo	1060–1260	UCD	1.07	-8.6	8.2	3.3
	5MT123-07	5MT123	Albert Porter Pueblo	1060–1260	UCD	1.08	-8.1	7.9	3.3
	5MT123-10	5MT123	Albert Porter Pueblo	1060–1260	UCD	0.86	-8.9	10.6	3.3
	5MT23-08	5MT23	Grass Mesa Village	725–920	WSU	1.42	-7.39	8.15	3.3
	5MT23-14	5MT23	Grass Mesa Village	725–920	WSU	1.49	-7.28	9.32	3.2
	5MT23-17	5MT23	Grass Mesa Village	725–920	WSU	1.36	-8.18	7.22	3.3
	5MT23-18	5MT23	Grass Mesa Village	725–920	WSU	1.38	-8.03	8.26	3.4
	5MT3807-01	5MT3936	Lillian's Site	1060–1260	UCD	1.21	-6.8	7.9	3.4
	5MT3807-13	5MT3936	Lillian's Site	1060–1260	UCD	1.18	-8.7	10.2	3.3
	5MT3807-14	5MT3936	Lillian's Site	1060–1260	UCD	1.17	-8.7	8.6	3.3
	5MT3936-01	5MT3936	Lillian's Site	1180–1225	UCD	0.85	-8.6	8.1	3.3
	5MT4475-01	5MT4475	McPhee Pueblo	725–980	WSU	1.25	-8.54	9.06	3.3
	5MT4475-08	5MT4475	McPhee Pueblo	725–980	WSU	1.43	-7.63	10.21	3.3
	5MT4475-12	5MT4475	McPhee Pueblo	725–980	WSU	1.46	-9.05	10.38	3.1
	5MT4475-19	5MT4475	McPhee Pueblo	725–980	WSU	1.49	-6.96	12.61	3.2
	5MT5-01	5MT5	Yellow Jacket Pueblo	1020–1280	UCD	1.24	-15.9	9.0	3.2
						**Average**	-9.1	9.1	
						**SD**	3.1	1.4	

Of the 62 dogs that yielded sequences, 56 were complete from nucleotide positions 15459–15691 (Table 2, S2 Table). Dog mtDNA sequences have been deposited in Genbank (accession numbers MF196981—MF197042). A Fisher’s exact test of haplotypes observed in the pre-1280 CE CMV and pre-1280 CE NRG provides no evidence of differentiation (p = 0.284) (Table 7). Since no dogs were identified in the post-1280 CE NRG samples, no comparison was made.

**Table 7:** P-values of Fisher’s exact tests used to compare haplotype counts for dogs across the three spatial/temporal units of analysis. Tests compare haplotype frequencies from Table 2; n/a = not applicable; tests could not be carried out because no dogs were observed in Post-1280 NRG samples.

	Pre-1280 CMV	Pre-1280 NRG
Pre-1280 NRG	0.284	
Post-1280 NRG	n/a	n/a

## Discussion

Our study confirms the observation of low mtDNA diversity among prehistoric, domestic Southwest US turkeys [22]. Importantly, our increased sampling of turkeys from some archaeological sites over those investigated by Speller et al. [22] (e.g., Shield’s Pueblo and Sand Canyon Pueblo (Table 1)), lends support to the notion that there are not frequent haplotypes beyond those initially described. No novel mtDNA lineages were identified in this study.

Turkey mtDNA haplogroup frequencies are consistent with the hypothesis that enough of these domestic birds were moved from the CMV (southwestern Colorado) to the NRG (north-central New Mexico) during the 13^th^ century CE to change the mtDNA haplogroup profile of the NRG population. The mtDNA composition of the turkeys maintained in the NRG prior to 1280 CE differs statistically from that of the turkey population post-dating 1280 CE (p = 0.0212), whereas post-1280 CE NRG turkeys are indistinguishable from those maintained in the CMV prior to 1280 CE (p = 1.0000). Notably, however, one matriline exhibited by two turkeys from the post-1280 CE NRG population (i.e., those exhibiting 15782T, 15793T, 15894T, 15953C (Table 1)) was exclusively observed from that unit of analysis and, thus, appears to have not arisen in the CMV prior to 1280 CE. As one specimen, AHT-21, dates to 1310–1345 CE, and the other dates to 1300–1845 CE [22], it is possible that it evolved in the post-1280 CE NRG turkey population. Future aDNA studies of turkeys from the region should be able to address this hypothesis.

It is also important to remember that previous studies have found that turkey remains are, in general, quite rare in NRG sites that pre-date 1200 CE, and that the incidence of turkey within the archaeological record increased substantially in the following century; concurrent with rapid human population growth [4:292–294, 43]. This archaeological pattern reinforces our finding that the turkey population of the post-migration NRG does not reflect an expansion of the pre-migration turkey population, but rather an influx of birds that accompanied human immigrants. Our results suggest the CMV was a plausible source area for both the turkeys and, by proxy, their human keepers.

Prehistoric dogs in the US Southwest exhibit a larger number of matrilines than turkeys. While seven matrilines were detected among the 56 dog samples, the most common lineage (i.e., 15484G, 15627G, 15639A) is exhibited by ~85.7% of them (forty-eight of fifty-six individuals). Interestingly, approximately 92.6% (one hundred and seventy-five of one hundred and eighty-nine; Table 1) of the turkeys belong to a single matriline. Given the strong genetic evidence for intensive management of turkey populations in the prehistoric US Southwest [18, 21, 22], it is possible to begin to evaluate a similar scenario for dog populations. Similar patterns of reduced genetic diversity have been observed from dogs recovered from archaeological sites in the US Southeast, and is interpreted as having arisen from deliberate prehistoric breeding practices [11]. Intensive dog breeding practices in the prehistoric US Southwest could also help to explain: 1) the differential diets ascertained for dogs studied here (Fig 2), and 2) the existence of two distinct-looking dog breeds (as ascertained from their mummified remains) recovered from White Dog Cave, Arizona dating to 400 BCE [44]. Future genetic analyses of these specific specimens and other mummified canids, especially in cases where the phenotype of “breed” can be ascertained, would be useful to ascertain whether matriline has relevance to breed, if at all.

As only coyotes were identified in the post-1280 CE Northern Rio Grande samples, possible changes in the NRG dog mitochondrial gene pool across this apparent migration boundary could not be addressed. However, unlike observations from turkeys, we found no evidence that pre-1280 CE dogs from the two regions are distinguishable (p = 0.284). This is primarily due to the genetic diversity of the sampled individuals, with a series of relatively rare haplotypes being represented. So it may be that turkeys, with their lower genetic diversity, are a better proxy for the identification of human population movements in any case.

The coyotes detected in our study appear to have been wild based on their stable carbon (^13^C/^12^C) isotopic values relative to the majority of dogs. Barring two dogs that have coyote-like δ^13^C values (samples 5MT123-03 and 5MT5-01 (Table 6 and Fig 2)), isotopic variation among coyotes is larger than among dogs. This suggests some degree of dietary plasticity with regards to trophic level among coyotes, a finding that is demonstrated in modern ecological studies of coyote as well [45, 46]. Coyotes are able to significantly alter their diet based on food availability and the presence of competitors. By contrast, domesticated dogs appear to have had a more homogenous diet, particularly with respect to accessing protein with C4-derived carbon. The smaller δ^15^N values of most dogs also indicate consumption of lower trophic-level foods, relative to coyotes. Why two dogs stand out isotopically and fall in the coyote cluster in Fig 2 is unclear, but will be the subject of additional analysis. Nevertheless, this demonstrates that genetic species identification of canid remains can improve respective species tabulations in archaeological reports. Genetically identifying species based on DNA would be critical if canid diets are to be used as “proxies” for human diets (which was not our aim here, but see e.g., Guiry [47]).

Overall, however, the findings are consistent with human management or breeding and dietary provisioning of dogs. This specific diet may have put selective breeding pressures on dogs as well. Specifically, dogs that responded well to the δ^13^C-enriched but δ^15^N-depleted diet would have been bred, while those that did not may have been culled. Given recent findings that domesticated dogs have many more copies of genes involved in the digestion of starches and fatty acids relative to wolves [48], we propose that the domestic canine diet included significant amounts of maize. In other words, dogs that responded well to a maize-rich diet were preferentially survived and were bred.

## Conclusions

Scholars have debated the role of migration in the collapse of ancestral Pueblo societies of the Four Corners region (including Southwest Colorado) and the coincident emergence of the Rio Grande Pueblos. Previous studies of biological variation using morphometric traits [3, 4:87–124] have found evidence of population movement from the CMV to the NRG during the 13^th^ century CE. Due to repatriation and native population discontinuity in the four corners region it is not currently possible to confirm these morphometric findings through analyses of contemporary or ancient human DNA. We have therefore considered mtDNA from remains of domestic species as a proxy for human population relationships. Domestic dogs and turkeys were sampled from three temporal/geographic groups for analysis: the pre-migration CMV (pre-1280 CE), the pre-migration NRG (pre-1280 CE), and the post-migration NRG (post-1280 CE).

Our analyses show that many of the canid remains were of coyotes, and isotopic analyses confirm that these coyotes consumed sources of carbon comparatively less enriched in ^13^C than those consumed by dogs. Although these results are tangential to our primary research question, they are still informative in that they are consistent with a scenario in which the dogs consumed a diet rich in maize, perhaps supplemented by some turkeys that also ate maize, whereas the coyotes likely subsisted on a wide range of wild resources rich in C3-derived carbon. As a result of no dogs being identified from post-migration NRG sites, we lack the necessary samples to test for discontinuity between pre-migration and post-migration NRG dogs. However, we were not able to detect statistically-significant genetic differences between pre-migration CMV and NRG dogs.

In contrast, our analyses of turkey mtDNA, combined with results of previous studies, provide evidence that the turkey population of the post-migration NRG exhibits genetic continuity with the pre-migration CMV turkey population, and both exhibit genetic discontinuity with the pre-migration NRG turkey population. These results are consistent with an influx of turkeys to the NRG during the 13^th^ century, and the CMV was not excluded as a possible source for these birds. They also suggest the post-1280 CE NRG turkey population could not have derived wholly from the pre-1280 population from this region. Our results are consistent with the hypothesis that migrating human populations from the CMV brought domestic turkeys with them to the NRG, leading to a change in the mtDNA composition of the post-1280 CE NRG turkey population. This study thus provides the first direct genetic evidence, by way of a domestic animal proxy, in support of the hypothesis that Tewa communities occupying the NRG region today derive in large measure from the migration of 13^th^ century CMV populations. If true, this would support a scenario in which migration was a primary solution of CMV populations in the face of significant social and environmental problems [2, 4, 49–51], as it often is for human groups under extreme duress today.

## Supporting information

S1 Table Turkey samples studied with their provenience, dates, and other pertinent archaeological information, and the full results of their genetic analyses. Under the amplicon columns, an “X” in a cell indicates successful amplification and sequencing of the amplicon, whereas “O” indicates results from the amplicon were not obtained. Partial sequences that could be used to confidently categorize individual turkeys as members of either the aHap1 or aHap2 lineage, but for which eventual resolution of these missing data may reveal them to belong to one or more derived lineages, are indicated with question marks as “aHap1?” or “aHap2?”. Sequence reads and mutational positions are relative to the turkey mtDNA reference sequence (EF153719; [30]).
(XLSX)

S2 Table Canid samples studied with their provenience, dates, and other pertinent archaeological information, and the full results of their genetic analyses. Under the amplicon columns, an “X” in a cell indicates successful amplification and sequencing of the amplicon, whereas “O” indicates results from the amplicon were not obtained. Cells colored black indicate that no attempt to amplify the amplicon was made. Dog sequence reads and mutational positions are relative to the dog mtDNA reference sequence (U96639; [34]). Coyote sequence reads and mutational positions are relative to the coyote mtDNA reference sequence (DQ480509; [35]).
(XLSX)
